# Obesity/overweight and asthma control in LEBANESE adults: a cross-sectional study

**DOI:** 10.1186/s12889-019-7116-3

**Published:** 2019-06-17

**Authors:** Carla Irani, Salim Adib, Georges Halaby, Abla Sibai

**Affiliations:** 1 Internal Medicine & Clinical Immunology Department, Hotel Dieu de France hospital, St Joseph University, Blvd A. Naccache, Beirut, 166830 Lebanon; 2 Division of Pulmonary Medicine, University of Alberta hospital, Alberta, Canada; 3 Faculty of Health Sciences, American University of Beirut, Beirut, Lebanon; 4 Endocrinology Department, Hotel-Dieu de France, St Joseph University Beirut, Beirut, Lebanon

**Keywords:** Asthma control test, Obesity/overweight, Asthma

## Abstract

**Background:**

Studies exploring the association between weight and asthma are not conclusive. Both obesity and asthma have been increasing in Lebanon, their association is not yet documented. The aim of this study is to explore the effect of weight on asthma control in adults.

**Methods:**

This is a cross-sectional study, involving all consecutive asthma patients presenting to the outpatient allergy clinic at the Hotel-Dieu de France (HDF) University Hospital between January 1, 2014 and December 30, 2016. Patients included were those who consented to fill the Asthma Control Test (ACT) after 3 months of therapy. BMI was reported at the same time of the questionnaire.

**Results:**

A total of 183 records of diagnosed asthma cases in adults were included. Sixty-three (34.4%) were males and 120 (65.6%) females, with a mean age of 38.5 (SD = 14.3). Ninety patients (49.2%) were of normal weight, 65 (35.5%) overweight and 28 (15.3%) obese. Seventy-one percent had an ACT score ≤ 19, which corresponds to poor asthma control. Patients who were overweight or obese were more likely to have poor asthma control compared to patients who had a normal weight at the time of evaluation.

**Conclusion:**

In conclusion, our study showed a significant association between asthma control as assessed by the ACT and high BMI defining overweight or obesity. This is the first national study exploring the association between asthma and overweight/obesity in Lebanon. A larger study with sampling from different specialists’ sites is needed to draw more conclusions about this association.

## Background

Asthma in adulthood is an increasingly detected respiratory disease, the control of which is often affected by various factors, including overweight. The association between weight and asthma control in adults has been based mostly on observational studies, and solid evidence is still needed. In Lebanon, obesity and asthma have been increasing but the actual concomitance of both health problems is not yet documented, while the negative impact of obesity on other chronic diseases such as diabetes and coronary vascular diseases has already been reported [1, 2].

The prevalence of asthma in Lebanese adults, expressed as the prevalence of hyper-reactive airways is estimated at 9% [3]. While effective asthma treatment is available, a meta-analysis including 300 million patients found that 20% of patients worldwide had severe illness despite adequate treatment [4]. The “Asthma Insights and Reality” study in Lebanon showed that about 20% of hospitalized patients had poorly controlled asthma [5]. The severity of asthma is usually classified according to the Global Initiative for Asthma (GINA) guidelines [6] as “controlled”, “partially controlled” or “uncontrolled”, based on detailed clinical definition. In observational studies, the contribution of overweight / obesity to the severity of asthma drew the attention of researchers. The prevalence of obesity in Lebanon is increasing: in 2009, the STEPS survey reported obesity in 26.1% of adults [7]. In 2008, the US National Asthma Survey suggested that obesity is associated with several measures of asthma severity and poor control, including symptoms, missed work days, medication use and GINA severity classification in adults [8]. Findings regarding the association between obesity and asthma control are not consistent. For example, it appears that the vulnerability of obese patients may vary with the allergic or non-allergic etiology of their asthma [9]. Some studies reject any potential link between Body Mass Index (BMI) and asthma control, and show that poorly controlled asthma may be associated with several socio-demographic factors [10]. The aim of this study is to explore the effect of weight on asthma control in adults in Lebanon.

**Fig. 1:**
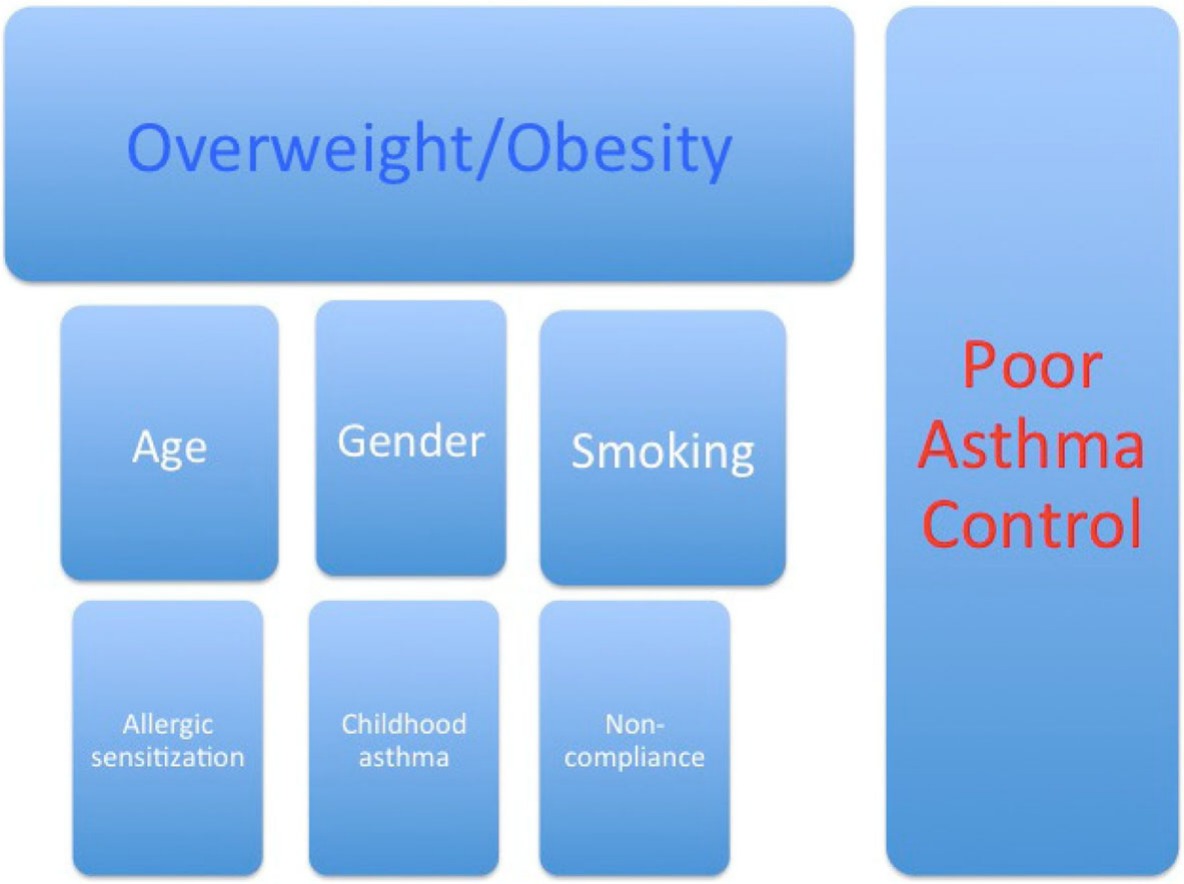
Conceptual model. A conceptual framework linking Asthma control to Overweight/Obesity in presence of possible confounders

A conceptual model is presented below to illustrate the research questions. Figure 1.

## Methods

This is a cross-sectional study involving all consecutive asthma patients presenting to the outpatient allergy clinic at the Hotel-Dieu de France (HDF) University Hospital between January 1, 2014 and December 30, 2016. Patients included were those who consented to fill a questionnaire evaluating disease control (Asthma Control Test: ACT) after 3 months of therapy. BMI was calculated after recording weight and height given by the patient when completing the questionnaire.

All included patients were 18 years of age and older when formally diagnosed with asthma according to the GINA classification, and followed-up exclusively at the HDF allergy clinic between January 1, 2014 and December 30, 2016. All participants should have received the same standard treatments as defined by the GINA guidelines [6]. The control of their asthma was evaluated within 3 months after the prescription of the treatment. According to the GINA guidelines, a clinical evaluation is done every 3 months to decide whether to step-up or step-down therapy. Patients were excluded if they presented with heavy co-morbidities: cancer, lupus or vasculitis diseases, congenital or acquired immunodeficiency, or chronic obstructive respiratory disease (COPD) (2% of the cross-sectional sample).

Overweight and obesity were defined using the Body Mass Index (BMI), as per the CDC standard definition [11]. Overweight represents a BMI of > = 25 kg/m^2^ and obese is a BMI > 30 kg/m^2^. Lower BMIs represent “normal weight”.

The outcome of interest was “asthma control” measured with the Asthma Control Test (ACT) that is specifically conceived to assess the level of asthma control in patients treated according to the GINA guidelines (GINA 2015) [12]. The ACT is a 5-item self-administered questionnaire previously validated in Arabic [13]. The sum of answers ranges from 5 to 25, with higher scores reflecting better asthma control. These scores are divided as follows: “uncontrolled” (scores 5–19) or “controlled” (20–24) asthma. The score of 25 indicates an optimal level of control [14]. For the purposes of this analysis, patients with an optimal score were included in the “controlled” group. Control variables belonged to 2 categories:

- Socio-demographic factors: age (≤45 and > 45 years old); gender; level of education (2 categories: undergraduate, post-graduate)
- Health-related factors: Tobacco use (2 categories: none ever, past/current smokers for tobacco and/or waterpipe); compliance with treatment (yes/no), history of allergic sensitization (measured by skin-test or specific respiratory IgE assay); history of childhood asthma (yes/no)

### Statistical analysis

Data analysis was performed on SPSS software version 23. A descriptive analysis was done using the counts and percentages for categorical variables and mean and standard deviation for continuous measures. The Student t-test was used to compare the means of continuous variables between two groups. For comparison between categorical variables, the chi-square and Fisher exact tests were used. Two backward logistic regressions were conducted, taking the good vs poor asthma control as the dependent variable and taking the BMI as a continuous variable in the first regression and taking it as a categorical variable in the second regression. All other variables that showed a *p* < 0.2 in the bivariate analysis were also taken as independent variables in the multivariable analysis models. A *P*-value less than 0.05 was considered significant in all cases.

### Ethical considerations

The project was approved by the Hotel-Dieu de France (HDF), St Joseph University ethical committee and the IRB committee at the American University of Beirut (AUB). The medical records examined were kept in a private storage area, and the chart review was done confidentially. Informed consent to contribute to research is a routine procedure at HDF, and only data from those who signed a consent were included.

## Results

A total of 183 records of diagnosed asthma cases in adults were available for analysis during the study period. Of those, 63 (34.4%) were males and 120 (65.6%) females, with a mean age of 38.5 (SD = 14.3). Based on the BMI, 90 (49.2%) patients were of normal weight at the time of evaluation of control by ACT, 65 (35.5%) were overweight and 28 (15.3%) were obese. For the analysis, we combined the overweight and obese. Only 13.1% were current smokers, and 76.5% had graduate or postgraduate degrees. Allergic sensitization was present in 79.2% of cases, and 68.3% did not report any childhood asthma. (Table 1).

**Table 1:** Sociodemographic and other characteristics of participants

Variables		Total *N* = 183 n (%)
Age		
18–45		110 (60.10)
> 45		73 (39.9)
Gender		
Females		120 (65.57)
Males		63 (34.42)
Graduate studies* (yes)		135 (73.77)
*Health related characteristics*		
Allergic sensitization** (yes)		147 (80.32)
Childhood asthma (yes)		58 (31.69)
Smoking status (cigarettes or waterpipe (Yes)		41 (22.40)
Compliance with treatment^ (yes)		117 (63.93)
*Clinical Profile*		
Body Mass Index (BMI) categories		
Normal weight (BMI < 25)		90 (49.2)
Overweight (25 ≤ BMI < 30)		65 (35.5)
Obese (BMI ≥ 30)		28 (15.3)

*Any graduate studies after high school

** Allergic sensitization confirmed by skin testing or specific IgE measurement

^Compliance is evaluated by number of refills for medication as well as direct interviewing

### Bivariate analysis

The results of the bivariate analysis, taking good vs poor asthma control as the dependent variable, showed that a significantly higher mean BMI was found in patients with a poor asthma control compared to those with a good asthma control (26.37 vs 24.03), whereas a significantly higher percentage of patients who were compliant to treatment had good asthma control compared to those who were non-compliant to treatment (66.04% vs 46.15%).

When dividing the BMI into categories, a significantly higher percentage of patients who had a normal BMI had good asthma control (78.8% vs 36.9%), whereas a higher percentage of patients who were overweight (43.8% vs 15.4%) or obese (19.2% vs 5.8%) had poor asthma control (Table 2).

**Table 2:** Bivariate analysis of the outcome “Poor Asthma Control” with covariates

Variables	Poor Asthma Control ACT≤19 n = 130	Good Asthma Control ACT> 19 n = 53	*p*-value*
Age category			
≤ 45 years old	88 (67.69)	42 (32.31)	0.193
> 45 years old	41 (77.36)	12 (22.64)	
Gender			
Females	90 (69.23)	30 (56.6)	0.103
Males	40 (30.77)	23 (43.44)	
Body Mass Index (continuous variable)	26.37 ± 3.94	24.03 ± 3.21	**< 0.001**
Body Mass Index categories			**< 0.001**
Normal (BMI < 25)	48 (36.9%)	41 (78.8%)	
Overweight (25 ≤ BMI < 30)	57 (43.8%)	8 (15.4%)	
Obese (BMI ≥ 30)	25 (19.2%)	3 (5.8%)	
Graduate studies (yes)	88 (67.69)	37 (69.81)	0.286
Allergy (yes)	102 (79.07)	44 (83.02)	0.486
Childhood asthma (yes)	41 (31.54)	17 (32.08)	0.944
Smoking (yes)	31 (23.85)	10 (18.87)	0.184
Compliance (yes)	60 (46.15)	35 (66.04)	**0.015**

*Numbers in bold indicate significant p-values

### Mutlivariable analysis

The results of a first logistic regression taking the good vs poor* asthma control as the dependent variable and taking the body mass index as a continuous variable, showed that a higher BMI (ORa = 0.839) was significantly associated with a poor asthma control. In addition, females (ORa = 0.531) had a poorer asthma control compared to their male counterparts, whereas being compliant to treatment (ORa = 1.848) was associated with a good asthma control (with the last two associations tending to significance) (Table 3, Model 1).

**Table 3:** Multivariable analysis

Variable	*p*-value	Adjusted odds ratio	95% Confidence Interval	
Model 1: Logistic regression taking the good vs poor^a^ asthma control as the dependent variable and taking the body mass index as a continuous variable.				
Gender (males^a^ vs females)	0.078	0.531	0.263	1.074
Body Mass Index	0.001	0.839	0.756	0.930
Compliance to treatment (no^a^ vs yes)	0.085	1.848	0.919	3.714
Model 2: Logistic regression taking the good vs poor^a^ asthma control as the dependent variable and taking the body mass index as a categorical variable.				
Compliance to treatment (no^a^ vs yes)	0.087	1.882	0.912	3.884
Body Mass Index overweight category compared to normal	< 0.001	0.155	0.062	0.389
Body Mass Index obese category compared to normal	0.002	0.131	0.035	0.485

^a^ Reference group

Variables entered in Model 1: age, gender, compliance to treatment, Body Mass Index

Variables entered in Model 2: age, gender, compliance to treatment, Body Mass Index categories

A second logistic regression taking the good vs poor asthma control as the dependent variable and taking the body mass index as a categorical variable, showed that being overweight (BMI between 25 and less than 30) (ORa = 0.155) or obese (BMI ≥30) (ORa = 0.131) compared to a normal BMI were associated with a poor asthma control. Being compliant to treatment (ORa = 1.882) was associated with a good asthma control, with this association tending to significance (*p* = 0.087) (Table 3, Model 2).

## Discussion

Increased BMI has been associated with poor asthma control in multiple studies, yet the association is complex and conflicting results are shown in different studies. The effect of age on poor asthma control in adults have shown that older age is usually associated with more severe asthma and poorer control because of the presence of other comorbidities. This was not the case in our study where younger age ≤ 45 had poorer disease control compared to older age. This may have been due to a selection bias since the mean age of our population is 38.5. This is most likely due to the fact that the population is selected from an allergy clinic where the population is in general younger. Indeed, in our study 80% of the patients showed aeroallergen sensitization, which is as well a selection bias. Allergic sensitization was not significantly associated with higher BMI or poorer asthma control: in both controlled and uncontrolled asthmatics, the percentage of allergic patients was much higher than the non-allergic. As far as gender, studies have shown that females are more likely to have poor disease control compared to males [15]. In our study, we had a higher percentage of females (65.57%) than males. Even though their association with poor asthma control was almost significant with females being more likely to have poorer control than males, the association with higher BMI was not statistically significant; indeed, we had almost the same number of female patients being of normal weight or overweight/obese. In the western world, it seems that females are more likely to be overweight [16]. Generally, this is not true in Lebanon where females are more concerned about their weight and body image [17]. Gender did not significantly affect the association between poor asthma control and BMI. The association with higher BMI was significant, where patients who have a graduate or postgraduate degree are more likely to have a higher BMI compared to patients who were undergraduates, but this association is still being studied to further strengthen this notion [18]. The association between graduate studies and poor asthma control was barely significant in the bivariate analysis of this study, even though almost 68% of patients who had graduate or postgraduate degrees, had poor asthma control as defined by the ACT. The presence of childhood asthma is not, in general, a risk factor for poor disease control in adulthood. There are no conclusive studies showing that the history of childhood asthma is associated with poor disease control in adults. Conversely, few studies have shown that a patient with a history of childhood asthma may be more aware of asthma management and therefore better controlled. Recent pediatric asthma guidelines include inhaled rather than oral corticosteroids for childhood asthma, therefore limiting the effect of treatment-induced weight gain in asthmatic. Our study showed no significant association between the history of childhood asthma with neither higher BMI nor poor asthma control. In our study, only 22% of our patients were cigarette or waterpipe smokers. This is a main selection bias and does not reflect the prevalence of cigarette or waterpipe smokers among the Lebanese population. One possible explanation may be that since we have a higher percentage of uncontrolled asthmatics, those prefers to avoid smoking since it will worsen their respiratory symptoms and exacerbate their asthma, although studies showed that allergic or asthmatic patients do not avoid smoking [19]. In our study, smoking was not associated with higher BMI, neither with poor asthma control. Again this may be due to a low percentage of smokers among our population. A study we previously conducted to explore the effect of parental smoking on asthma exacerbation of their children showed that only 28% of parents were smokers [20] .

Adherence to asthma therapy is the subject of numerous publications in developed countries [21]. In general, adherence to therapy may vary across cultures but it is a fact that non-compliance to therapy is associated with poor asthma control [22]. Therefore, medical authorities in developed countries devote a lot of resources on asthma education in an attempt to overcome the factors associated with non-adherence. Among those factors are: patients’ beliefs and goals, lack of time given by physicians to explain and supervise inhaler techniques, economic problems and much more. Social desirability bias should be taken into account, because the treating physician or his clinic assistant assessed adherence to therapy. In our study, compliance was found to be present in approximately 64% of cases; there was no association with higher BMI, but it seemed that non-compliant patients have poor asthma control.

This study has several limitations. First, it is cross-sectional, thereby introducing a potential selection bias. Another limitation of this study design is that it can investigate an association between overweight/obesity and poor asthma control but it cannot prove causal mechanisms. Since the study population is from a single allergy clinic, this may lead to selection bias. However, clinical information was collected at the time of the clinic visit, thus reducing recall bias. The last limitation is the fact that the follow-up was done at 3 months after the initiation or the adjustment of the therapy, our plan is to extend the follow-up over a longer period.

## Conclusion

In conclusion, our study showed a significant association between poor asthma control as assessed by the Asthma Control Test and high BMI defining overweight or obesity. A larger study with sampling from different specialists’ sites is needed to draw more conclusions about this association.

## Data Availability

“The datasets used and/or analysed during the current study available from the corresponding author on reasonable request.”

## Abbreviations

ACT: Asthma Control Test
AUB: American University of Beirut
BMI: Body Mass Index
CDC: Centers for Disease Control
GINA: Global Initiative on Asthma
HDF: Hotel Dieu de France
